# Waveguide-Enhanced Raman Spectroscopy (WERS): An Emerging Chip-Based Tool for Chemical and Biological Sensing

**DOI:** 10.3390/s22239058

**Published:** 2022-11-22

**Authors:** Pengyi Wang, Benjamin L. Miller

**Affiliations:** 1 Departments of Biomedical Engineering, University of Rochester, Rochester, NY 14642, USA; 2 Departments of Dermatology, University of Rochester, Rochester, NY 14642, USA; 3 Departments of Biochemistry and Biophysics, University of Rochester, Rochester, NY 14642, USA; 4 Institute of Optics, University of Rochester, Rochester, NY 14642, USA; 5 Materials Science Program, University of Rochester, Rochester, NY 14642, USA

**Keywords:** photonics, spectroscopy, Raman, sorbent polymers

## Abstract

Photonic chip-based methods for spectroscopy are of considerable interest due to their applicability to compact, low-power devices for the detection of small molecules. Waveguide-enhanced Raman spectroscopy (WERS) has emerged over the past decade as a particularly interesting approach. WERS utilizes the evanescent field of a waveguide to generate Raman scattering from nearby analyte molecules, and then collects the scattered photons back into the waveguide. The large interacting area and strong electromagnetic field provided by the waveguide allow for significant enhancements in Raman signal over conventional approaches. The waveguide can also be coated with a molecular class-selective sorbent material to concentrate the analyte, thus further increasing the Raman signal. This review provides an overview of the historical development of WERS and highlights recent theoretical and experimental achievements with the technique.

## 1. Introduction

There is an ever-increasing need for rapid yet low-cost detection of small molecules in many fields including point-of-care (POC) clinical diagnostics, environmental and industrial surveillance, and safety monitoring for food and other consumer goods. Sensors, defined here as devices that measure the presence and/or concentration of biological or chemical analytes, are already playing an important role in addressing these needs [1]. Most biosensing techniques with high specificity require covalently attached probes (antibodies, enzymes, DNA/RNA strands, aptamers, etc.) to act as specific biorecognition element for analytes [2,3,4,5]. Both “labeled” immunoassay techniques such as lateral flow assays and novel formats including lab-on-a-chip and label-free biosensors widely employed in POC detection [6] rely on the high affinity of antibodies toward their respective antigens for analyte recognition. However, the use of biopolymeric capture probes is less feasible when the molecular weight of the target analyte is small, in part because of the small binding that surface small molecules present (preventing the use of sandwich antibodies or labeling reagents). For label-free biosensors reliant on refractive index-based changes, small molecules present an additional challenge because they do not perturb the response of the sensor unless at high concentration. In the context of POC diagnostics, the most prominent set of diagnostically relevant molecules is the basic metabolic panel (BMP), which consists of glucose, calcium, sodium, potassium, carbon dioxide, chloride, BUN (blood urea nitrogen), and creatinine. These analytes range in molecular weight from 23 to 113 g/mol. Likewise, many of the analytes of interest industrially and in the context of environmental monitoring are small molecules.

**Figure 1:**
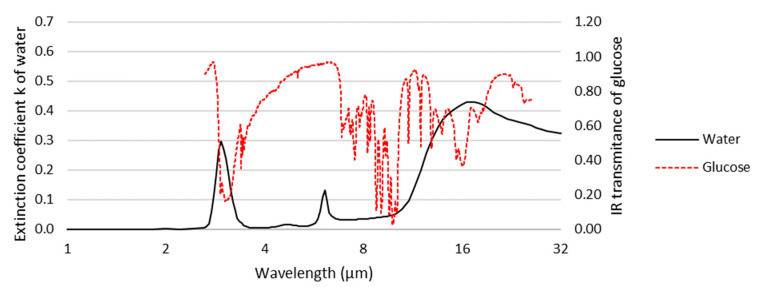
Extinction coefficient of water superimposed on the infrared spectrum of glucose (using data adapted from [12,13]). Water strongly absorbs at wavelengths corresponding to several of the characteristic bands of glucose, hindering its observation in aqueous samples by infrared spectroscopy.

To simplify the detection of small molecules, considerable research effort is being focused on vibrational spectroscopies, including infrared (IR) and Raman spectroscopy, as they directly identify and quantify molecules using vibrational characteristics uniquely determined by their molecular structure. Surface IR spectroscopy with various enhancement mechanisms, mainly attenuated total reflection infrared (ATR-IR) spectroscopy [7,8,9], infrared reflection absorption spectroscopy (IRAS or IRRAS) [10], and surface-enhanced infrared spectroscopy (SEIRAS) [8,9], have been used in the biosensing of small molecules [11]. However, since biological samples of interest are usually aqueous, IR spectroscopies suffer from the strong absorption of water over their mid-infrared (MIR, ~2–16 μm) operating wavelength (Figure 1). It is for this reason that Raman spectroscopy provides distinct advantages.

Raman spectroscopy detects the inelastic Raman scattering of incident photons by analyte molecules [14]. The inelastically scattered photons have a wavelength shift (also known as a Stokes shift) from the wavelength of the incident photons, which is a function of the vibrational energies of the molecule undergoing analysis. By choosing an appropriate original wavelength of incident light (typically described as the “pump wavelength”), the scattered Raman signal can avoid the strong absorption wavelength range of water or other substrates. While this provides a distinct advantage over absorption spectroscopy, Raman spectroscopy also has a significant disadvantage in that Raman scattering is intrinsically very weak. For example, Rohleder et al. compared mid-IR and Raman spectroscopy in the quantitative analysis of serum and found that similar accuracy was achievable with both techniques, but the measurement time needed for Raman spectroscopy was 10 times longer (5 min) than for mid-IR spectroscopy (30 s) [15]. To address this problem, efforts have been made to enhance the scattering. Surface-enhanced Raman scattering (SERS) uses metal (mostly silver and gold) nanostructured substrates to locally enhance Raman scattering from analyte molecules by factors up to 10^6^–10^10^ [16,17]. Despite many successful applications of SERS in biosensing [16,18,19] the reproducibility and robustness of the substrate are continuing concerns [17,20]. Moreover, SERS requires the analyte molecules to fall in optimally enhancing “hotspots” around the metal nanostructures, which may be difficult and inefficient when the analyte concentration is low and can also further increase the inconsistency of the detection. The hotspots may also get blocked by non-analyte molecules in complex samples [14].

WERS is an alternative, recently emerging [21] approach to enhancing the sensitivity of Raman that does not rely on metal nanostructures, thereby avoiding the challenges of SERS discussed above. WERS relies on the interaction of analytes with the evanescent field of a waveguide (discussed below) on a photonic chip. In contrast to SERS, where Raman scattering only comes from the enhancing hotspots, in WERS, analytes can interact with the evanescent field over the entire length of the waveguide, and the waveguide also collects and conducts the Raman scattering to the spectrometer. In theory, waveguides provide dozens-fold enhancement of Raman signal per cm [22]. For example, a direct experimental comparison of WERS with a standard confocal Raman microscope reported by Dhakal et al. [23] achieved more than four-orders-of-magnitude higher spontaneous Raman signal for WERS. As we discussed below, different configurations and the use of polymer sorbents can provide enhancements well beyond these levels (up to 10^8^). The manufacture of waveguides for integrated photonics applications is mature and continuously improving because it relies on the same processes used in the microelectronics industry [24]. Waveguide structures can be produced with high consistency, avoiding the manufacturing scalability concerns that remain a challenge with SERS. Of course, as a label-free spectroscopic technique, there is also no need for perishable and expensive labels or probes.

### WERS Basics

**Figure 2:**
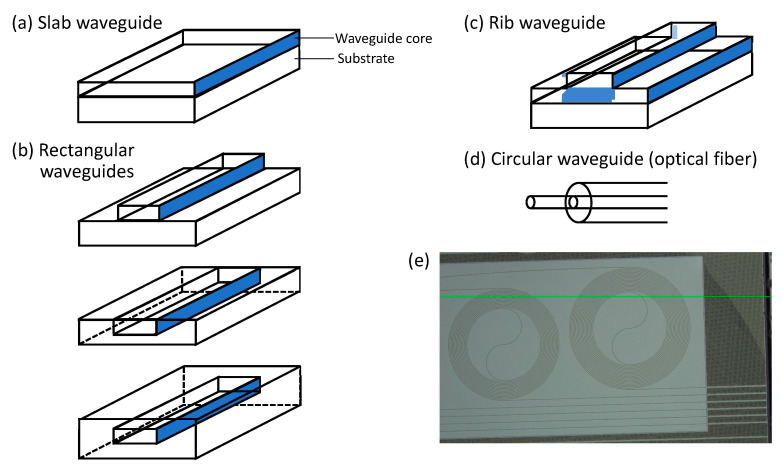
(**a**–**d**), adapted from [25]) Basic waveguide structures: slab waveguide, rectangular (channel/strip/wire) waveguide, and circular waveguide (optical fiber). Slot waveguides, not shown in this figure, consist of two rectangular waveguides positioned closely together in parallel. (**e**) Laser confocal microscope image of a WERS chip with spiral waveguides. The green line is a horizontal imaging reference.

A dielectric optical waveguide is a structure that conducts electromagnetic waves, i.e., light. Waveguides constrain light and allow it to propagate along a “core” with higher refractive index compared to surrounding “cladding”, via total internal reflection. For example, an optical fiber is a type of waveguide. In this case, the core is glass (silica) or a polymer, and the cladding is either a polymer with a lower refractive index than the core or air. Figure 2 shows some common types of optical waveguides. Waveguides are useful in sensing in large part because the electromagnetic field of light conducted along the waveguide is not totally confined in the core region. It extends into the cladding, producing what is called an evanescent field. Light can interact with molecules falling in the evanescent field and cause Raman scattering. Early experiments with WERS used the electromagnetic field in the core, whereas the majority of recent efforts make use of the evanescent field for sensing. Our discussion of the development of WERS covers both cases but emphasizes the latter.

## 2. Historical Development of WERS

As mentioned above, WERS was first demonstrated with liquid-core waveguides [26]. In an early report, a 10 to 25 m hollow fused quartz optical fiber with core diameter of ~75 μm was filled with benzene (C_6_H_6_) or tetrachloroethylene (C_2_Cl_4_) as the analyte. The waveguide conducted the pump laser and collected the Raman signal from the analyte core. Compared to conventional Raman spectroscopy, this technique enhanced the signal by factors of 10^2^ to 10^3^.

While such early experiments using optical fibers for WERS were impressive, implementation of the technique in a chip-based format paved the way for compact, low-cost sensing systems with significant advantages of size, weight, and power (SWAP) typical of integrated photonics [27]. A key demonstration of WERS by Levy in 1974 used a slab waveguide consisting of the analyte itself [28]. Methyl methacrylate (MMA) was made into a film a few μm thick and served as the core, both conducting the pump laser light and collecting the Raman signal of the material. Five years later, Rabolt [29] pushed the thickness of analyte thin film to as small as 1 μm without apparent background Raman from the substrate holding the film. These efforts demonstrated the feasibility of WERS, but also highlight the need to minimize Raman signals from the waveguide itself if one wants to detect an analyte external to the waveguide.

As a next step up in complexity from the analyte-core waveguides described above, Rabolt et al. [30] tested waveguides with a two-layer core. Two films together served as the core: one film was a thicker, known material with a well-characterized Raman spectrum, while the other film was only a few nm thick and actually the analyte to be tested. In this way, the analyte could be a molecular monolayer, and this Raman signal reflects the information about the bonds between the analyte and the thicker layer. In some ways, the analyte in this implementation effectively straddles the core and evanescent regions of the waveguide.

Similarly structured waveguides were used to measure the Raman signal of 8 nm polystyrene, a monolayer of the protein bovine albumin and dimyristoyl phosphatidylethanolamine (DMPE) Langmuir–Blodgett monolayers [31]. The substrate, core, and cladding were SiO_2_, ZnO, and air, respectively. The analyte was a thin film directly on the top of the ZnO core; however, here, it was considered to be located in the evanescent field instead of serving as a part of the core. The ZnO has higher refractive index than the polymer and biological analytes; thus, the local electric field intensity is higher and better enhances the Raman scattering. That is the reason why using a high-refractive-index core and exciting the analyte with the evanescent field became the mainstream of WERS. However, since the core is also exposed to the electromagnetic field, a potential disadvantage of this approach is that its emission will also be collected. Here, two peaks in the Raman spectrum of the ZnO core obscure the weak signal from analyte around their wavelength range. This result highlighted the need to optimize the material of the core to prevent core Raman signals from interfering with analyte signals.

For the example discussed above, Raman signals were collected using a lens positioned above the waveguide rather than at the waveguide end, as would be required for a fully integrated solution. In a follow-up to their work with ZnO core waveguides, the Greve group tested Si_3_N_4_ under the hypothesis that this would produce significantly less background as a core material than ZnO [32]. Here, the measurement geometry was also changed; this time they collected the Raman signal from the waveguide output. It was previously demonstrated both experimentally [33] and theoretically [34] by O’Connor and Tauc that Raman scattering excited by the evanescent field in the waveguide cladding can couple back to the guided mode. Although the original purpose of this finding was to bring notice to the fact that the optical fibers used in Raman spectroscopy generate background from the fiber’s core and cladding, it also laid the groundwork for later WERS studies using the evanescent field for sensing.

As hypothesized, Greve and colleagues found Si_3_N_4_ to have favorable characteristics for waveguide-based Raman measurements. This waveguide material has become the most commonly used waveguide materials for WERS, given its good collection efficiency and low background [35]. It is also convenient because of parallel developments in photonic integrated circuits (PICs) and in the computer industry. Complementary metal–oxide–semiconductor (CMOS) is a type of transistor which is critical for constructing electronic integrated circuit chips such as microprocessors and memory chips. Increasing the density of CMOS gives the chips higher computing power or bigger storage capacity. For years, Moore’s law has successfully predicted that technology developments in the industry would drive a doubling in the number of transistors on an electronic chip every 12–24 months. However, transistor scaling has reached a critical size limit due to electron leakage [36]. Thermal management has also become increasingly challenging as the density of electronic circuitry on computer chips has increased. Photonic circuits have the potential to overcome the bottlenecks imposed on electronic integrated circuits and continue pushing the boundaries of Moore’s law [37]. The fabrication process of photonic circuits can be executed with the CMOS fabricating techniques [38]. This CMOS compatibility is a key driver for the growth of PICs in other areas, including sensing.

**Figure 3:**
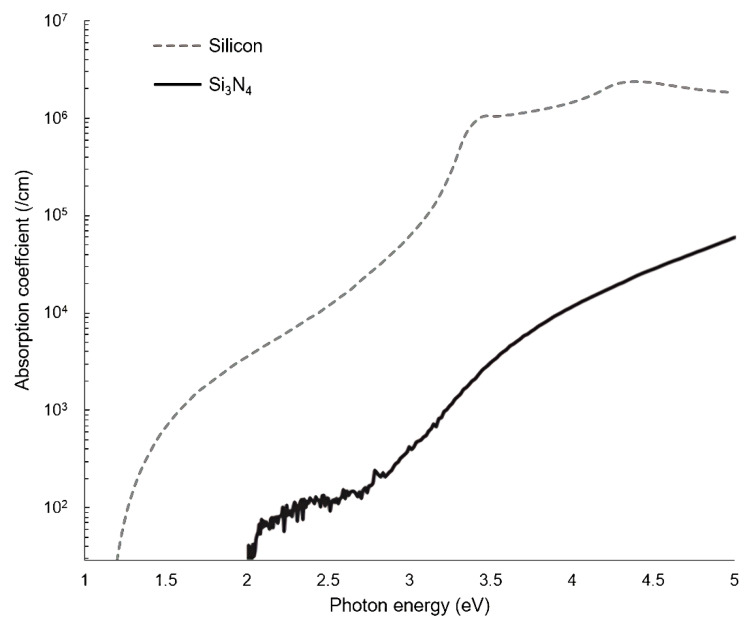
Absorption coefficient for Si_3_N_4_ and silicon as function of incident photon energy (E = *h*c/λ, where *h* is Planck’s constant, c is the speed of light, and λ is the wavelength; the x-axis is equivalent to 1240–250 nm; using data adapted from [43,44]).

Silicon-on-insulator (SOI) and silicon nitride (SiN) are the two most common materials used in the production of PICs, and they are compatible with standard CMOS fabrication methods [39]. As such, there is a large manufacturing infrastructure developed by the microelectronics industry that can be leveraged for PIC production. Standard SOI and SiN platforms are typically around 200–220 nm thick, although significant customization is possible. This design convention for waveguides can be tailored according to the specific needs of the application. Compared to SiN, SOI is theoretically usable but much less preferred for WERS due to silicon’s strong absorption at wavelengths typically used for WERS [23]. The intensity of Raman scattering is proportional to the fourth power of the excitation frequency [40]; hence, the wavelength chosen for the pump laser is as short as possible to maximize the intrinsically small Raman signal. This must be balanced against the fluorescence and absorption of the waveguide material, which increase at shorter wavelengths. Comparing the absorption coefficients of SiN (only showing Si_3_N_4_ here) and silicon (Figure 3), SiN has a significant advantage in this regard. However, the fluorescence/luminescence of SiN waveguides can be a problem; therefore, it is important to understand what contributes to the background luminescence of Si_3_N_4_ waveguides and how this can be reduced. Dhakal et al. [41] reported that SiN luminescence originates primarily from two sources: the narrow-band peak component is from impurities, especially hydrogen-based compounds and interstitial nitrogen, while the broad-band slowly varying component is from thermal fluctuations of the refractive index and momentum selection rule breaking in the amorphous material. For the slowly varying component, the authors suggested that, if the material is more crystalline, the background spectrum may contract to narrower wavenumber ranges. Those findings remind us to pay attention to the details of waveguide fabrication, such as choosing appropriate chemical vapor deposition and annealing methods. Continued development of manufacturing processes have allowed both loss and luminescence in silicon nitride waveguides to be reduced to manageable levels [42].

## 3. WERS Theory for Rectangular/Channel/Strip/Wire Waveguides

The enhancement factor of WERS versus conventional Raman spectroscopy has been calculated as early as in the first WERS paper by Levy et al. [28]. However, these calculations focused on WERS using the field in the core, instead of the evanescent field in the cladding to generate Raman scattering. With further development of WERS for general-purpose chemical and biological sensing, the most common design is one in which the analyte molecules are located in the evanescent field outside the waveguide, which makes the calculation much less straightforward.

To assess the theoretical performance of WERS for biosensing, Jun et al. [45], Dhakal et al. [46], and Stievater et al. [22] first developed a theoretical framework for the excitation, collection, and enhancement of Raman signal with Si_3_N_4_ waveguides, before testing the theory experimentally. The emitting molecule is modeled as an oscillating dipole near the waveguide surface. In the discussion below, we present the key aspect of WERS theory, drawing from [22,45,46].

Collection efficiency:

In the weak coupling regime, the spontaneous emitting rate $\gamma_{WG}\left({\vec{r}}_{e},\omega \right)$ of a dipolar (analyte) molecule can be calculated using Fermi’s golden rule [45].

(1) $$\gamma_{WG}\left({\vec{r}}_{e},\omega \right)=2\pi {\left|g\left({\vec{r}}_{e},\omega \right)\right|}^{2}D\left(\omega \right),$$

where ${\vec{r}}_{e}$ is the dipole position, ${\left|g\left({\vec{r}}_{e},\omega \right)\right|}^{2}$ is the coupling strength between the dipole and the waveguide mode (expressed as electromagnetic field E), and D(ω) is the density of states (DOS).

For a single-mode rectangular waveguide, we can assume a 1-D DOS due to single-polarization operation. Since we only consider propagation in one direction (i.e., from source to detector along the waveguide), the 1-D DOS should be divided by 2:

(2) $$D\left(\omega \right)=\frac{l}{2\pi v_{g}\left(\omega \right)},$$

where l is a unit length and will be canceled out later.

Assuming the dipole is oriented parallel to the electric field, the coupling strength term in Equation (1) is given by the following [45]:

(3) $${\left|g\left({\vec{r}}_{e},\omega \right)\right|}^{2}={\left|{\vec{d}}_{0}\cdot \alpha \vec{E}\left({\vec{r}}_{e}\right)/ћ\right|}^{2}=\omega {\left|{\vec{d}}_{0}\right|}^{2}/\left(2ћ\varepsilon \varepsilon_{0}{\tilde{V}}_{eff}\right),$$

where $d_{0}$ is the dipole. The normalization factor α is given by the following [45]:

(4) $${\left|\alpha \right|}^{2}=ћ\omega /\iint \left\{\varepsilon_{0}\frac{d\left(\varepsilon \omega \right)}{d\omega }{\left|E\right|}^{2}+\mu_{0}{\left|H\right|}^{2}\right\}d\vec{r}\approx ћ\omega /\iint 2\varepsilon_{0}\varepsilon {\left|E\right|}^{2}d\vec{r}.$$

The effective mode volume ${\tilde{V}}_{eff}$ is defined as follows [45]:

(5) $${\tilde{V}}_{eff}={\tilde{A}}_{eff}\cdot l,$$

where l is the same unit length as in Equation (2). Lastly, the effective mode area ${\tilde{A}}_{eff}$ at the dipole position ${\vec{r}}_{e}$ is the following [45]:

(6) $${\tilde{A}}_{eff}\left({\vec{r}}_{e}\right)=\frac{\iint \varepsilon_{0}\varepsilon \left(\vec{r}\right){\left|E\left(\vec{r}\right)\right|}^{2}d\vec{r}}{\varepsilon_{0}\varepsilon \left({\vec{r}}_{e}\right){\left|E\left({\vec{r}}_{e}\right)\right|}^{2}}=A_{eff}\cdot \frac{\max\left[\varepsilon_{0}\varepsilon \left(\vec{r}\right){\left|E\left(\vec{r}\right)\right|}^{2}\right]}{\varepsilon_{0}\varepsilon \left({\vec{r}}_{e}\right){\left|E\left({\vec{r}}_{e}\right)\right|}^{2}}.$$

Equations (1)–(6) describe the spontaneous emitting rate $\gamma_{WG}\left({\vec{r}}_{e},\omega \right)$. However, since the emitted light decays in all directions including as it approaches the waveguide, the decay rate also needs to be considered. According to Novotny and Hecht [47] and Jun [45], the decay rate $\gamma_{free\;space}\left(\omega \right)$ in the background medium is

(7) $$\gamma_{free\;space}\left(\omega \right)=\frac{\omega^{3}\sqrt{\varepsilon }{\left|d_{0}\right|}^{2}}{3\hbar \pi \varepsilon_{0}c^{3}}.$$

Plugging Equations (1)–(7) together, the effective Raman cross-section (collection efficiency), which is the portion of the emission power that can be collected into the waveguide, is

(8) $$\sigma_{eff}=\frac{P_{WG}}{P_{free\;space}}=\frac{\gamma_{WG}\left({\vec{r}}_{e},\omega \right)}{\gamma_{free\;space}\left(\omega \right)}=\frac{3}{8\pi }\frac{c}{nv_{g}}{\left(\frac{\lambda_{0}}{n}\right)}^{2}\frac{1}{{\tilde{A}}_{eff}}.\;$$

Excitation efficiency and overall efficiency:

Power radiated by the dipole $d_{0}$ in free space is expressed as follows [46]:

(9) $$P_{free\;space}=\frac{\omega^{4}{\left|d_{0}\right|}^{2}}{12\pi \varepsilon_{0}c^{3}},$$

where

(10) $${\left|d_{0}\right|}^{2}=\alpha^{2}{\left|E\left({\vec{r}}_{0}\right)\right|}^{2}\frac{n_{g}P_{pump}}{\iint c\varepsilon_{0}\varepsilon \left(\vec{r}\right){\left|E\left(\vec{r}\right)\right|}^{2}d\vec{r}}.\;$$

This describes the excitation of the dipole by the evanescent field. Plugging Equations (9) and (10) into Equation (8), the overall efficiency combining both excitation and emission $\frac{P_{WG}}{P_{pump}}$ can be calculated.

Coupling efficiency and waveguide length:

In reality, coupling of the pump laser light into the waveguide and the Raman signal out of the waveguide are not perfect. To take these coupling losses into account, $P_{pump}$ and $P_{WG}$ need to be modified by efficiency terms $\gamma_{in}=\frac{P_{pump}}{P_{in}}$ and $\gamma_{out}=$ $\frac{P_{out}}{P_{WG}}$, where $P_{in}$ is the laser power and $P_{out}$ is the collected Raman power. Therefore, for unit length and a single dipole as the analyte molecule,

(11) $$\frac{P_{out}}{P_{in}}=\gamma_{in}\gamma_{out}\frac{P_{WG}}{P_{pump}}.$$

In addition to coupling efficiency, there is also a loss during light traveling along the waveguide. Longer waveguides provide a larger contacting area with analyte molecules but suffer more from propagation loss. Modifying Equation (11) with the effect by waveguide length, the final efficiency becomes the following [46]:

(12) $$\frac{P_{out}\left(L\right)}{P_{in}}=\rho \gamma_{in}\gamma_{out}\frac{P_{WG}}{P_{pump}}\int_{0}^{L}e^{-\alpha_{p}z}e^{-\alpha_{s}\left(L-z\right)}dz=\rho \gamma_{in}\gamma_{out}\frac{P_{WG}}{P_{pump}}e^{-\alpha_{p}L}\left(\frac{e^{\Delta \alpha L}-1}{\Delta \alpha }\right),$$

where ρ is the molecule density, L is waveguide length, $\alpha_{p}$ and $\alpha_{s}$ are the propagation losses at the pump wavelength and at the Stokes wavelength, respectively, and $\Delta \alpha =\alpha_{p}-\alpha_{s}$.

## 4. Experimental Verification of WERS Theory

Equation (12) was verified by Dhakal et al. [46] using rectangular Si_3_N_4_ waveguides with different lengths. Although the results obtained were found to have a significant standard deviation, the experimental data were consistent with the equation. Likewise, work from the Stievater lab demonstrated good correlation between theory and experiment, but also some variability due to variation in waveguide fabrication and other factors [22]. Later, Dhakal et al. [48] validated the equation for both TE and TM mode using waveguides with different lengths, widths, designs (rectangular and slot waveguides), and refractive indices (Si_3_N_4_, TiO_2_, and silicon). According to their results, for rectangular Si_3_N_4_ waveguides, the fundamental quasi-TM mode has an efficiency nearly 2.5 times larger than the quasi-TE mode, and this difference gets larger for waveguides with higher refractive indices. The quasi-TE mode of slot waveguide can achieve even larger efficiency than both TE and TM for a rectangular waveguide. These findings provide direction for researchers seeking to improve their waveguide design for WERS.

## 5. Raman Emission Collected at the End of Waveguide vs. at the Surface of Waveguide

The theory developed above is focused on collecting Raman signals at the end of a waveguide. As seen in our discussion of early WERS experiments with slab waveguides, collecting from the top surface is also a possibility, and this has the advantage of not needing careful coupling of light from the waveguide into an optical fiber or microscope lens. To test the relative efficiencies of these two methods, Wang et al. [49] compared the collection of Raman emission from the waveguide end and the surface with a 110 nm Ta_2_O_5_ slab waveguide. Because of the geometry of the slab, the pump light was chosen to be in TM mode. The dipole under the electric field, therefore, oscillates perpendicular to the waveguide surface. In this case, they calculated that 76.3% of the Raman emission from a dipole will couple into the waveguide. For the remaining proportion, if a coverslip is put above the waveguide to reflect some of the escaping light back into the waveguide, the light coupled will increase by 17.8%. Moreover, collecting at the end accumulates the Raman signal from the analyte molecules all along the waveguide, whereas collecting at the surface only covers a relatively small area. Thus, for better enhancement, the collection site should be at the end, assuming that the propagation loss and coupling loss are not so large as to cancel the benefit. Rectangular waveguides have a substantially smaller top surface area than slab waveguides, which have a width considered to be infinite relative to the wavelength of light. As such, the mode profile of a rectangular waveguide is more similar to that of an optical fiber, making the coupling at the end of the waveguide more efficient.

## 6. Silicon Nitride and Other Materials for WERS Sensing

**Figure 4:**
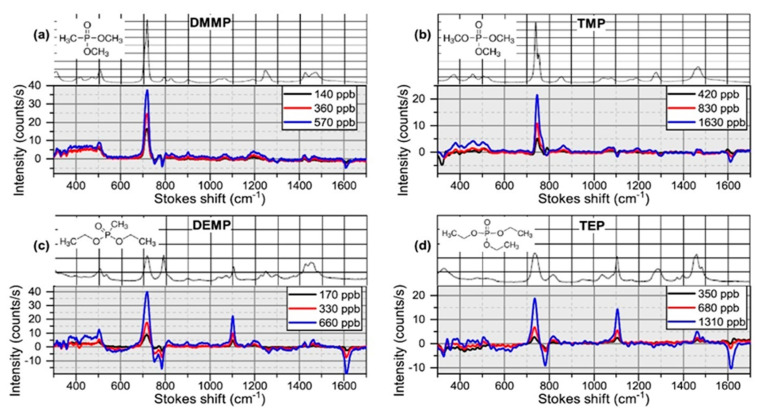
(Reprinted with permission from [50] © Optica) Background-subtracted WERS spectra of (**a**) DMMP, (**b**) TMP, (**c**) DEMP, and (**d**) TEP at different concentrations compared to their reference Raman spectra.

Having discussed the early history of and theory behind WERS, we now turn to examples of where Si_3_N_4_ WERS has been put to use. Tyndall et al. [50] used WERS with Si_3_N_4_ rib waveguides to measure the trace concentrations of four vapor-phase chemical warfare agent simulants: dimethyl methylphosphonate (DMMP), diethyl methylphosphonate (DEMP), trimethyl phosphate (TMP), and triethyl phosphate (TEP). The waveguide core was as thin as 110 nm (compared to a 175 nm core waveguide studied for TE mode) and etched by 55 nm to create the rib structure. A carbosilane sorbent polymer which absorbs the four analyte molecules was deposited on top of the waveguide. This hyperbranched hydrogen bond acidic polymer semi-selectively binds and concentrates the hydrogen bond basic analytes to enhance their Raman signal. The polymer’s background spectrum was taken to make sure it did not have Raman or fluorescence peak interfering with the analytes’ peaks. The thin core was found to decrease the core background and increase the overlap of the quasi-TM mode with the sorbent cladding, improving the signal-to-noise ratio. Figure 4 shows that the height of the Raman peak from the analyte is proportional to the concentration, suggesting that the technique can be made at least semi-quantitative.

In this example, the sorbent layer was key to reaching a low limit of detection. The idea of using solid-phase absorbents to preconcentrate the analyte, thus increasing its Raman signal, was first raised in 1997 by Ewing et al. [51]. The development of analyte class-selective sorbents remains a significant opportunity for extending the capabilities of WERS. In addition to capturing and concentrating analytes of interest, sorbent polymers can provide spectral simplification of a complex mixture by rejecting molecules outside the structural class for which they are designed. The physical barrier provided by a sorbent may prove to be particularly important in whole-blood analysis; hemoglobin present in red blood cells, as well as other blood pigments, produces a large background fluorescence signal that is a well-known complication of Raman measurements in blood [52]. As a guide to evaluating polymer sorbents for WERS, Tyndall et al. described an experimental framework, including figures of merit [53].

**Figure 5:**
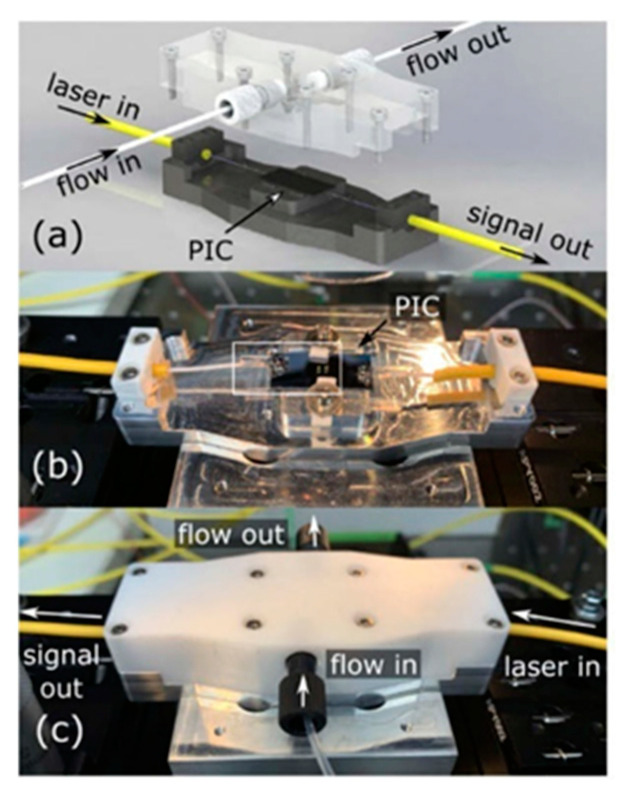
(Reprinted with permission from [54] © Optica) Fiber-packaged, microfluidic-coupled WERS. (**a**) Rendering of the assembly with optical fibers affixed to the chip and a flow cell that lowers onto the chip surface. (**b**) Photograph of the packaged chip with fibers glued to the edge. (**c**) The packaged chip with the flow cell secured on the top.

For WERS PICs to be useful outside of specialized research laboratories, they must be packaged with optical fibers for light I/O and with microfluidics for sample delivery. Kita et al. [54] reported a packaged and fiber-bonded WERS chip. The coupling fibers were aligned and glued to the chip, and the flow cell for analyte sample was then secured above (Figure 5). A notable feature of the experimental design of this work is that data were collected in a back-reflection Raman signal, allowing the signal to be separated from the forward-propagating pump and waveguide background.

**Figure 6:**
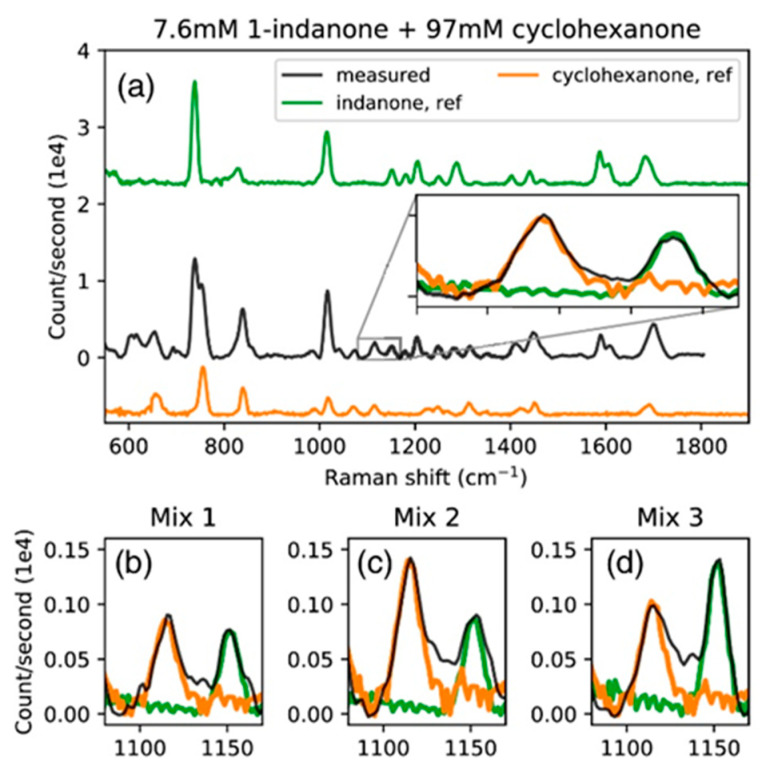
(Reprinted with permission from [55] © Optica) Background-subtracted spectrum of (**a**) 7.6 mM (1 g/L) 1-indanone and 97 mM (1%) cyclohexanone solution, (**b**) 1.9 mM (0.25 g/L) 1-indanone and 24 mM (0.25%) cyclohexanone, (**c**) 1.9 mM 1-indanone and 48 mM (0.5%) cyclohexanone, and (**d**) 3.8 mM (0.5 g/L) 1-indanone and 24 mM cyclohexanone.

Liu et al. [55] demonstrated a WERS sensor incorporating a Si_3_N_4_ slot waveguide for detection of low-concentration organic pollutants in water. Mesoporous silica modified by hexamethyldisilazane (HMDS) was used as top cladding to absorb and enrich pollutants in the aqueous sample by 600-fold, which further enhanced the sensitivity of the device. Figure 6 shows the sensor simultaneously quantifying multiple analytes. After measurement, the analyte could be desorbed in 3 min by putting the sensor in pure water. This regeneration ability makes the sensor reusable and reduces the cost.

In addition to Si_3_N_4_, Ta_2_O_5_ has been studied as a material for the waveguide core. Due to its high refractive index contrast with SiO_2_, Ta_2_O_5_ waveguides confines the mode more tightly than Si_3_N_4_ does, enabling the development of more compact photonic circuits with smaller bending radii. Another advantage is that the pumping laser wavelength for Ta_2_O_5_ waveguides is in the visible range, making alignment easier and safer. Of course, these advantages must be balanced against the fact that the manufacturing infrastructure for Ta_2_O_5_ is much less developed than that for silicon nitride.

Coucheron et al. [56] presented a WERS sensor based on a Ta_2_O_5_ slab waveguide for blood analysis. They chose 532 nm as the pump laser wavelength to measure the resonance Raman signal from hemoglobin. Resonance Raman typically focuses only on detection of a single analyte, since the photon energy of the pump laser needs to match the electronic absorption band of the analyte. The authors began with a 15.5 mM solution of hemoglobin in distilled water, allowing the solution to dry on the waveguide measurement. Of course, blood is a highly complex matrix, and significant work remains to demonstrate routine, quantitative detection of analytes in blood using WERS. However, this work constitutes a “foot in the door” for WERS in the field of blood testing.

Ta_2_O_5_ also may be combined with glass to make composite optical waveguides. Hu and Qi [57] used such a waveguide for a sensor with a mixed concept of SERS and WERS to overcome the reproducibility challenges often seen with SERS. A 10 nm thick copper phthalocyanine (CuPc) film, used as both the cladding and the analyte, was deposited on the waveguide surface. A solution of NaCl was flowed over the film to increase the refractive index, such that the mode shifts toward the cladding and the electromagnetic field intensity at the CuPc film increases. This effect enhances not only the Raman signal from the film but also the absorption of the pump light by the film. This double enhancement provided an eightfold enhancement of the Raman signal in TM mode. The authors suggested that this approach could be a useful method for monolayer and biomembrane analysis.

As mentioned previously, the waveguide core material needs to have low absorption and emission at the pump wavelength and over the spectral range of Raman signal from analytes of interest. Meanwhile, the refractive index of the core material affects the mode distribution; more specifically, it determines the fraction of the electromagnetic field that can be evanescent and interact with analytes. Raza et al. [58] made a comparison of Al_2_O_3_, Si_3_N_4_, Ta_2_O_5_, and TiO_2_, which are suitable as the core for WERS considering their high transparency through near-IR wavelengths and the ability to conduct high-intensity laser light. They found that, because of high refractive index contrast versus the cladding, TiO_2_ (n = 2.33) had the best signal collection efficiency. Si_3_N_4_ (n = 1.89) and Ta_2_O_5_ (n = 2.11) had comparable collection efficiency, and Al_2_O_3_ (n = 1.60) had the worst efficiency among the four materials. However, Al_2_O_3_ had the lowest background emission, whereas TiO_2_ had the highest, eight times higher than Si_3_N_4_ and Ta_2_O_5_. After balancing the signal collection efficiency and the background, the authors believed Si_3_N_4_ and Ta_2_O_5_ stood out, but the more highly mature fabrication capabilities for Si_3_N_4_ led to a lower waveguide loss, thus representing the current optimum choice.

In addition to these four common materials, Makela et al. [59] used aluminum nitride (AlN) waveguides fabricated with a CMOS-compatible process for WERS. They chose AIN because it has a suitable refractive index (n = 2.1), and the background can be an order of magnitude lower than silicon nitride in the visible regime. Since Raman intensity is proportional to the fourth power of the excitation frequency, using a pump laser of visible light instead of IR increases the Raman signal. The 550 nm thick slab AlN waveguide was fabricated on a 3 μm thick SiO_2_ undercladding. A 635 nm pump laser was butt-coupled into the waveguide in TM mode. Analyte solution was dropped on the surface, and the Raman signal was collected above the waveguide. Although collecting from above is not as efficient as collecting at the end of waveguide (as discussed above), the authors were able to detect benzene derivatives (toluene and anisole) in benzene as a proof of concept.

**Figure 7:**
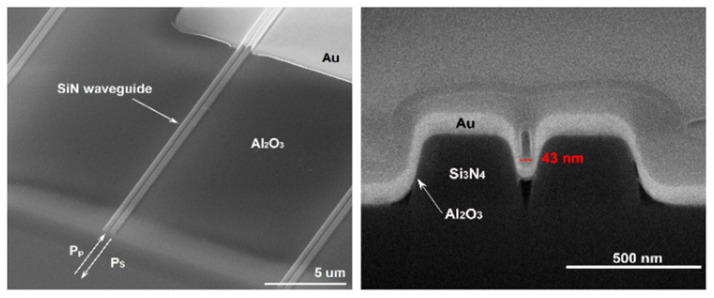
(Reprinted with permission from [61] © Optica) Waveguide-based nanoplasmonic slot waveguide top view and cross-section.

Lastly, using a composite core material, an intriguing combination of WERS and SERS was described by the Baets group [60,61]. They covered Si_3_N_4_ slot waveguides first with Al_2_O_3_ to narrow the slot, and then with Au to serve as an extended enhancing hotspot (Figure 7). Although the Raman signal intensity detected by this setup is weaker compared to conventional SERS, it generates a lower background and, thus, provides a similar signal-to-noise ratio to conventional SERS [58]. The substrate of waveguide-based SERS, i.e., the covered waveguide, is reproducible and consistent. Given that the Raman signal is collected from a waveguide, this implementation is convenient for lab-on-a-chip applications.

## 7. Summary

While still at a relatively early stage as a field of research, WERS is already demonstrating its utility as a sensing approach. By allowing for excitation of analyte molecules along an entire wavelength (which may reach 10’s of cm in a total area <1 cm^2^), WERS provides significant sensitivity enhancements relative to the intrinsically low signals of “standard” Raman spectroscopy. The evanescent field can be optimized by waveguide geometry to increase the intensity and by refractive index contrast to increase its overlap with analyte. The escaped Raman emission can even be reflected back to the waveguide with additional components such as a coverslip or the boundary of cladding layer. The ability to further enhance detection capability via the use of sorbent coatings, which may be analyte class-selective in addition to providing concentration enhancement, is a further advantage. Lastly, the small footprint and compatibility with CMOS fabrication of WERS are attractive characteristics.

Waveguide loss and background represent two significant technical challenges to WERS. Losses include propagation loss and coupling loss. Bends and imperfections in waveguide fabrication such as rough walls contribute to the propagation loss and can be improved by design and fabrication techniques. Coupling loss can be minimized by matching the mode profile between the waveguide and the other optical component, which is usually an optical fiber or a lens. Edge couplers have been intensively studied for this purpose [62]. The background is determined by the waveguide material. During design, researchers should select the waveguide material whose emission peaks are far away from the Raman signal of analytes. During fabrication, appropriate procedures also lower the background by reducing the impurity and amorphism. Recent results from various foundries indicate that the loss and background challenges can be overcome. The performance of the waveguide can also be affected by the environment. For example, the refractive index of Si_3_N_4_ is temperature-dependent [63]. This requires either that the WERS setup sits in a room with a constant temperature or controlling module, or that it gets calibrated prior to use.

Of course, integration of additional components of a WERS system (source, filters, waveguides, and spectrometer) may be considered. For example, Tyndall et al. [64] demonstrated the successful integration of an on-chip filter for WERS to get rid of the pump laser before spectrum collection. The cascaded Mach–Zehnder interferometer lattice filter blocked 75% of the power at the notch wavelength and maintained >90% transmission for wavelengths outside the filter notch. Considerable insight is also being gained into on-chip sources and spectrometers, although this is outside the scope of this manuscript. In addition to filters, rapid advances in the integrated photonics field are allowing for the integration of light sources and other even more complicated components on chips together with the waveguides. These developments give WERS a bright future in sensing.

## List of Abbreviations

AlN	Aluminum nitride
ATR-IR Spectroscopy	Attenuated total reflection infrared spectroscopy
BMP	Basic metabolic panel
BUN	Blood urea nitrogen
CMOS	Complementary metal–oxide–semiconductor
CuPc	Copper phthalocyanine
DEMP	Diethyl methylphosphonate
DMMP	Dimethyl methylphosphonate
DMPE	Dimyristoylphosphatidylethanolamine
DOS	Density of states
HMDS	Hexamethyldisilazane
IR	Infrared
IRAS or IRRAS	Infrared reflection absorption spectroscopy
MIR	Mid-infrared
MMA	Methyl methacrylate
PICs	Photonic integrated circuits
POC	Point-of-care
SEIRAS	Surface-enhanced infrared spectroscopy
SERS	Surface-enhanced raman scattering
SiN	Silicon nitride
SOI	Silicon-on-insulator
SWAP	Size, weight, and power
TEP	Triethyl phosphate
TMP	Trimethyl phosphate
WERS	Waveguide-enhanced raman spectroscopy
